# Insights into the angiogenic effects of nanomaterials: mechanisms involved and potential applications

**DOI:** 10.1186/s12951-019-0570-3

**Published:** 2020-01-09

**Authors:** Wenjing Liu, Guilan Zhang, Junrong Wu, Yanli Zhang, Jia Liu, Haiyun Luo, Longquan Shao

**Affiliations:** 1 Stomatological Hospital, Southern Medical University, Guangzhou, 510280 China; 2 Guangdong Provincial Key Laboratory of Construction and Detection in Tissue Engineering, Guangzhou, 510515 China

**Keywords:** Nanomaterials, Blood vessel, Endothelial cells, Angiogenesis, Tissue regeneration, Angiogenic property

## Abstract

The vascular system, which transports oxygen and nutrients, plays an important role in wound healing, cardiovascular disease treatment and bone tissue engineering. Angiogenesis is a complex and delicate regulatory process. Vascular cells, the extracellular matrix (ECM) and angiogenic factors are indispensable in the promotion of lumen formation and vascular maturation to support blood flow. However, the addition of growth factors or proteins involved in proangiogenic effects is not effective for regulating angiogenesis in different microenvironments. The construction of biomaterial scaffolds to achieve optimal growth conditions and earlier vascularization is undoubtedly one of the most important considerations and major challenges among engineering strategies. Nanomaterials have attracted much attention in biomedical applications due to their structure and unique photoelectric and catalytic properties. Nanomaterials not only serve as carriers that effectively deliver factors such as angiogenesis-related proteins and mRNA but also simulate the nano-topological structure of the primary ECM of blood vessels and stimulate the gene expression of angiogenic effects facilitating angiogenesis. Therefore, the introduction of nanomaterials to promote angiogenesis is a great helpful to the success of tissue regeneration and some ischaemic diseases. This review focuses on the angiogenic effects of nanoscaffolds in different types of tissue regeneration and discusses the influencing factors as well as possible related mechanisms of nanomaterials in endothelial neovascularization. It contributes novel insights into the design and development of novel nanomaterials for vascularization and therapeutic applications.

## Introduction

Incurable tissue defect is a challenge in clinical practice. The commonly used autologous or allograft tissue transplantation methods for tissue repair pose a series of problems, including immunological rejection, donor deficiency and other surgical risks. Tissue engineering is an emerging research field that has good application prospects and avoids the above limitations. However, insufficient vascularization has become a challenge hindering the clinical application of tissue engineering.

Blood vessels are distributed throughout the body, transport nutrients and oxygen, and remove carbon dioxide and waste to meet the various needs of physical activities. Thus, early vascularization is a hallmark for tissue repair, such as bone regeneration, skin wound healing and ischaemic tissue reperfusion. Angiogenesis involves the activation and migration of vascular endothelial cells, which form a new vascular network based on existing capillaries and/or venules [1]. The vascular network also accelerates the growth and reconstruction of surrounding tissues by providing a local microenvironment through immune modulatory mechanisms or paracrine signal release [2]. Therefore, targeting angiogenesis is a major promising therapeutic strategy for successfully constructing biomimetic tissue [3, 4].

Currently, tissue engineering strategies to enhance vascularization can be roughly divided into three common types of methods; the first type involves the loading of vascular growth factors [5]. However, due to the short half-lives and instability of vascular growth factors, there could be complications in biomedical applications. The second type involves in vivo vascularization by co-culturing with endothelial cells [6]. However, the cellular activity and utilization rates are low. The lack of standards for the culture and transplantation of seed cells increases the complexity of clinical translational medicine. The third type uses micro-engineering technology for vascularized mesh implantation; nonetheless, there are still some problems, such as differences in the organizational integration of blood vessels using microsurgical techniques. Therefore, it is necessary to improve the accuracy of vascular network reconstruction and the integration of the mesh with tissue [7]. Biomaterial scaffolds are considered a key component in tissue engineering. They construct the basic framework of the tissue structure, thus affecting the biological behaviour of cells as well as the release and efficiency of growth factors. Fully utilizing the properties of the biomaterials and achieving well-designed scaffold structures are important tasks for promoting functional angiogenesis and further tissue regeneration and remodelling.

Nanomaterials refer to materials with nanometre scales in at least one dimension. With the development of nanotechnology, nanomaterials have exhibited good application prospects for the early detection, diagnosis and tissue engineering application due to their unique physical and chemical properties and quantum size effects [8, 9]. The importance of nanomaterials to promote angiogenesis in tissue regeneration has received more and more attention [10]. However, different physicochemical properties of nanomaterials and their dominant roles promoting angiogenesis in tissue engineering have not been summarized and analysed. Nanoparticles could be endocytosed into immune cells or endothelial cells via clathrin and caveolae, causing changes in cellular behaviours that facilitate angiogenesis [11]. Nanofibres, electrospun scaffolds, or other mesoporous structure nanoscaffold materials can mimic the natural extracellular matrix (ECM) of blood vessels, which is beneficial for the adhesion, proliferation, and migration of endothelial cells and vascular endothelialisation [12, 13]. In addition, nanomaterials can also serve as delivery vectors to improve the sensitivity and targeting of proangiogenic factors [14].

Herein, we summarize the application of nanoscaffolds in tissue regeneration along with their angiogenic effects with regard to their physicochemical properties to regulate endothelial cell behaviour. The possible angiogenic potential of nanomaterials and the related mechanisms involved in different stages during angiogenesis are further discussed to provide guidance for future research.

## The application of nanomaterials promotes angiogenesis in tissue regeneration

Promoting early angiogenesis is a necessary factor for development and tissue regeneration. Due to the existence of tissue specificity, such as differences in the strength, elastic modulus and three-dimensional structure in hard and soft tissues, cardiovascular tissues and nerve tissues, the biomaterials used in tissue engineering should compliant with the structural and functional requirements of the target tissues. Scaffolds can not only provide support for tissue growth but also effectively introduce cells and growth factors into the defect site. Therefore, the angiogenic effects of nanoscaffolds involved in different types of tissue repair will be introduced below (Table 1).

**Table 1:** Biomedical application of nanomaterials in promoting neovascularization

Application	Nanomaterials	Type of angiogenesis assays	References
Bone engineering	Nano-HA	(1) In vitro study (2) In vivo glucocorticoid-induced bone defect model	[28]
		In vitro study	[29, 33, 154, 155]
		In vivo ectopic osteogenesis study	[18]
		In vivo calvarial defect model	[20, 99]
	Micro/Nano-structured surfaces of Cu_x_-HA	(1) In vitro study (2) In vivo subcutaneously implant study	[34]
	TCP nanolayers	In vitro study	[21]
	Nanofibrin	In vitro study	[24]
	GO	In vivo calvarial defect study	[22, 26]
		In vivo ectopic osteogenesis study	[27]
	PCL nanofibrous biomembranes	In vivo maxillary bone lesion model	[16]
	Mesoporous bioactive glass nanoparticles	(1) In vitro study (2) In vivo ectopic osteogenesis study	[30]
	Copper doped in electrospun bioactive glass nanofibers	In vitro study	[17]
	Micro-nano bioactive glass particles	In vitro study	[31]
	Mesoporous silica nanoparticles	In vitro study	[32]
Soft tissue wound healing	Gold nanoparticles	(1) In vitro study (2) In vivo wound model	[14, 38, 43, 44]
	Nano-sized bioactive glass	(1) In vitro study (2) In vivo wound healing assay	[39]
	Cerium oxide nanoparticle	(1) In vitro study (2) In vivo wound model	[42]
	the PCL nano-composite membranes incorporated with Zn-doped hollow mesoporous silica nanospheres	(1) In vitro study (2) In vivo wound model exposed to Escherichia coli	[40]
	PLLA electrospun fibrous membranes	(1) In vitro study (2) In vivo diabetic wound model	[35]
	Cu_2_S Nanoflowers	(1) In vitro study (2) In vivo diabetic wound model	[36]
	CaCuSi_4_O_10_ nanoparticles coated on the surface of Poly (ε-caprolactone) and Poly (D, L-lactic acid) (PP) fibers	(1) In vivo diabetic wound model cancer surgery-caused wounds in tumor-bearing mice	[37]
Nerve tissue repair	rGO	In vivo spinal cord hemisection model	[48]
	PLGA nanoparticles	In vivo spinal cord hemisection model	[45]
Ischemia reperfusion	GO	(1) In vitro study (2) In vivo myocardial infarction model	[49, 50]
	Tetrahedral DNA nanostructures	In vitro study	[137]

*HA* hydroxyapatite, *TCP* tricalcium phosphates, *GO* graphene oxide, *rGO* reduced graphene oxide, *PLLA* Poly-l-Lactide, *PLGA* poly (lactic-co-glycolic acid), *PCL* polycaprolactone

### Bone tissue engineering

The vascular network that forms at a bone defect facilitates the migration, differentiation and bone formation of osteoprogenitor cells not only through the supply of oxygen and nutrients but also through the interactions between endothelial cells and osteocytes. When vascularization is disrupted, bone formation is delayed and reduced [15]. The failure of osteogenesis after implantation in vivo is mainly due to a lack of angiogenesis in the defect area. Angiogenesis-related factors, such as vascular endothelial growth factor (VEGF) and hypoxia inducible factor (HIF) 1α, can significantly promote osteoblast differentiation and osteogenesis. Thus, effective vascularization is essential for promoting bone defect repair and functional restoration [16–18].

Several different biomaterials for bone tissue regeneration have been extensively studied, but single-scaffold materials cannot meet the requirements of good biocompatibility, vascular regeneration, new bone formation and the mechanical properties at the same time [19]. Therefore, researchers are working on fabricating novel micro-nano scaffolds to drive angiogenesis and promote bone regeneration [20]. The common method is infiltration of nanoparticles, nanosheets or nanofibres in different natural or synthetic materials, such as bioceramics [21, 22], polycaprolactone [23], chitosan [24], silk fibroin [25] and collagen [26–28]. The composition of nanomaterials improves the mechanical properties and surface hydrophilicity of the bone tissue engineering scaffold, which is beneficial to the growth and adhesion of human umbilical vein endothelial cells (HUVECs) [29]. The introduction of nanofibrin promotes the formation of neovascularization and avoids the cost of using large amounts of fibrin [24]. Nano-bioactive glass can also be added to the scaffolds for bone tissue engineering. Compared with microcrystalline bioactive glass, nano-bioactive glass can not only obtain a higher specific surface area and three-dimensional channel structure but can also increase the release of silicon ions and calcium ions to promote osteogenesis and angiogenesis [30, 31].

In addition to their nanostructures or chemical properties, which can affect the cytoskeleton or produce biological effects of angiogenesis, nanoscaffolds can also be used as carriers of small molecules or proteins with pro-angiogenicity, such as deferoxamine, adrenomedullin, VEGF, and other molecules [32, 33]. Such scaffolds not only reduce the toxicity of their direct action on endothelial cells but also coordinate the release of multiple growth factors. The use of such scaffolds is more efficient and stable than the addition of these factors directly to the matrix. The direct incorporation of ionic components with angiogenesis is also a strategy to promote vascularized bone tissue engineering scaffold modification. Copper is an important trace element in the human body. It can upregulate the expression of VEGF and promote the proliferation of endothelial cells. The flower-like micro-/nanostructured hydroxyapatite scaffolds were fabricated in solutions containing copper ions under hydrothermal conditions, which are beneficial to the proliferation of endothelial cells in vitro and for stimulating angiogenesis in vivo [34]. However, research on this aspect is limited; thus, the characteristics and slow-release structure of its specific ions are still unclear.

An ideal scaffold for bone tissue engineering should promote vascularized bone formation. Despite the continuous emergence of new scaffolds designed to optimize angiogenesis and promote osteogenesis, most research studies have simply focused on the histological manifestations of angiogenesis. However, the crosstalk and spatiotemporal dynamics underlying osteogenesis and angiogenesis have not been fully elucidated. Osteoblasts can secrete VEGF, which are conducive to angiogenesis. However, the viability of osteoblasts would be reduced due to a lack of nutrient exchange. Therefore, the use of effective design to promote the establishment of an early blood supply is an urgent challenge for bone tissue engineering scaffolds.

### Skin wound healing

Skin is the basic barrier to protect tissues from external damage and to maintain fluid balance. Therefore, technology for accelerating wound healing has important clinical significance. Promoting angiogenesis and restoring tissue perfusion can meet the metabolic requirements of inflammation, re-epithelialization and collagen matrix deposition. Synthesizing the ECM with similar elasticity, tensile strength and compressibility is also a key step in the skin wound healing. Moreover, the presence of open wounds and exudates, or the presence of diabetes or skin cancer, makes the stability and cost of biomaterial application even more challenging.

Nanoscaffolds have become a reliable research domain for wound healing therapies due to their biomedical properties, such as hydrophilicity, interactions with biological targets and deeper tissue penetration, making them a potentially ideal technology. Electrospinning mimics the native ECM and can be extensively used to produce nanofibrous scaffold. On one hand, stress is supported by embedded nanofibres, so that the elasticity of matrix is reinforced. On the other hand, they have been demonstrated to improve the adhesion and proliferation of endothelial cells and promote angiogenesis [35–38].

Nano-bioactive glass and mesoporous silica nanospheres fabricated on a nanofibre membrane can enable the greater release of silicon ion, promote the proliferation and migration of endothelial cells and fibroblasts, and upregulate the expression of genes related to angiogenesis and new tissue formation [39, 40]. The extracellular vesicle-mimicking nanovesicle hydrogel fabricated for the localized delivery of LncRNA-H19, which is an important target for triggering angiogenesis, has shown good therapeutic effects in diabetic wounds [41].

Gold nanoparticles and cerium oxide nanoparticles as metal-based nanopaticles deposited in a scaffold exhibit intrinsic proangiogenic activities that are beneficial for wound treatment [42–44]. In addition, Lino has developed a light-responsive plasmonic gold nanocarrier that can deliver two types of miRNA (miR302a and miR155) and sequentially regulate cell proliferation and human outgrowth endothelial cell survival, thereby promoting wound healing [14]. The combination of gene therapy with nano-delivery systems has attracted increasing attention due to the low antigenicity and higher efficiency of this method.

Wound healing is a complex physiological process that involves preventing infection, restoring perfusion, re-epithelialization and collagen fibre remodelling. Compared with traditional wound healing methods, nanomaterials have the potential to escape degradation by wound proteases, crossing bacterial biofilms and cell barriers into the cytoplasmic space to have protective biological effects in poor wound conditions (e.g., high glucose concentration or tumour). However, the current research has not systematically explored the changes in the physicochemical properties and biocompatibility of nanomaterials in various cell types where skin barrier damage occurs, and the research on targeted delivery to wound sites is limited. While fully exploiting the potential of nanomaterials, the preparation process is complex, which exposes the limitations of their clinical applications.

### Nerve tissue repair

The nanomaterial itself can not only regulate the formation of synapses but also integrate with nerve cells to regulate biological functions. Thus, nanomaterials are ideal scaffold materials for nerve injury repair and can be used as a bridge to transmit signals between nerve cells.

Blood vessels and nerves are closely related to each other. During development, blood vessels and nerves parallel each other and share a common regulatory mechanism. Nerves play an important role in the maturation and regulation of vascular function, and blood vessels provide growth factors, such as VEGF [45], for the growth and development of nerves. Vascular lesions, such as peripheral arterial lesions due to ischaemia, affect the nerve function of upper and lower limb conduction [46]. Acute myocardial ischaemia stimulates the innervation of primary sensory nerves and the sympathetic and parasympathetic nervous systems, enhancing the upregulation of substance P and inducing local inflammation [47]. Enhancing the capacity for angiogenesis in nerve tissue engineering offers the potential to repair segmental nerve defects. An acellular spinal cord scaffold with poly (lactic-co-glycolic acid) nanoparticles encapsulating VEGF_165_ was shown to promote angiogenesis and myelination in a rat spinal cord hemisection model [45]. The implantation of 3D scaffolds composed of reduced graphene oxide (GO) revealed some regenerated neuronal axons and new blood vessels [48], suggesting that a proangiogenic property of GO plays a role in nerve regeneration.

The interaction between nerves and vascular tissue has been increasingly considered, but its coordination and optimization in nerve tissue repair have not been discussed in depth. The angiogenic role of nanomaterials in nerve regeneration is worthy of further study, especially in nerve innervation functional recovery.

### Ischaemia reperfusion

Cardiovascular diseases are the main threat to human health. Stimulating the regeneration of blood vessels in the ischaemic area is an important way to reconstruct the cardiovascular system and restore its function. The use of mesenchymal stem cells (MSCs) alone or the local injection of some paracrine factors simply resulted in a poor local effect.

**Fig. 1:**
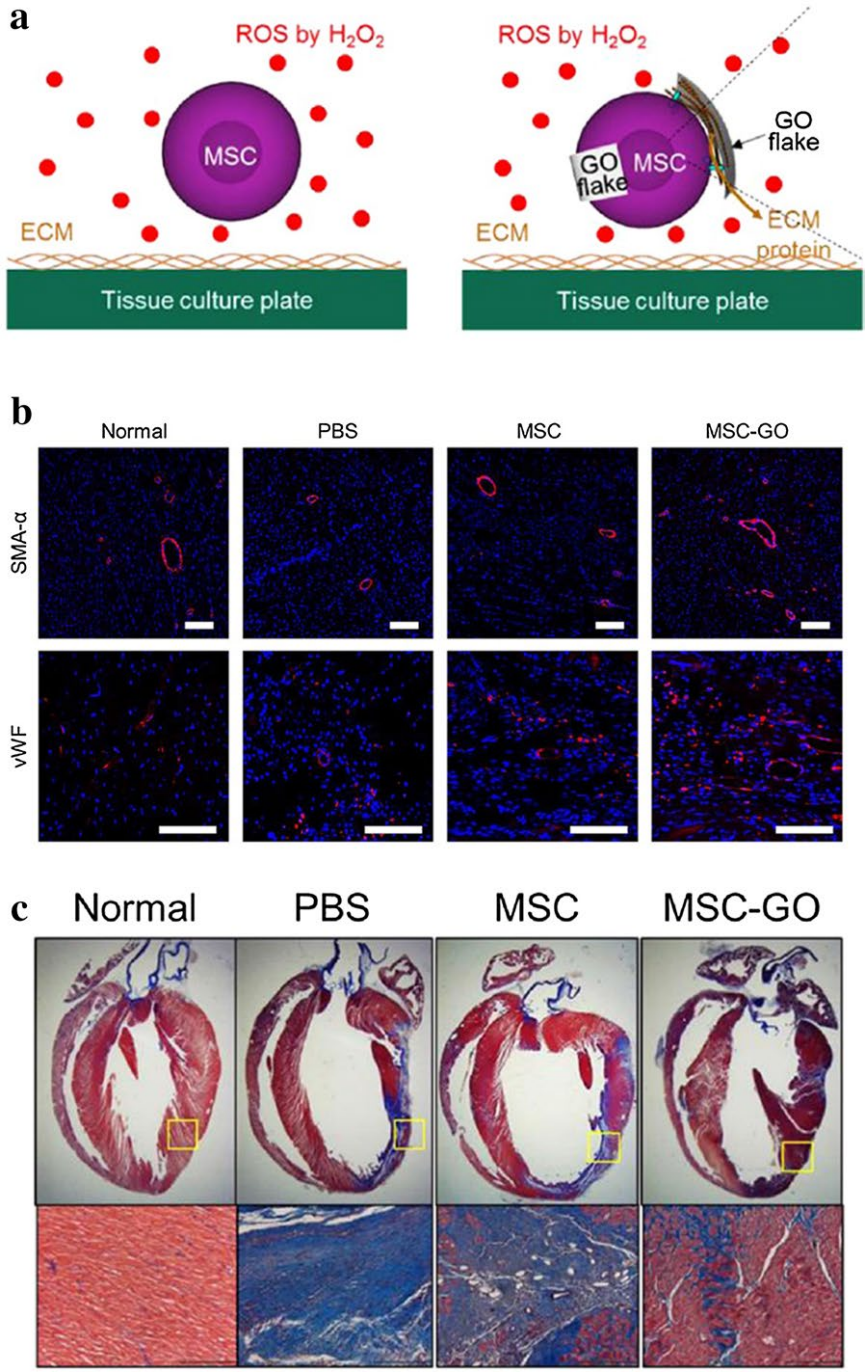
The therapeutic efficacy of the MSC or MSC-GO injected into the infarcted myocardium. **a** MSC adhesion to GO flakes avoids ROS-mediated deterioration in cell adhesion. **b** Microvessel density in the infarcted region 14 days after MSC or MSC-GO implantation. **c** Enhanced cardiac repair by MSC-GO implantation. Reprinted with permission from [50]. Copyright 2015 American Chemical Society

The introduction of various nanomaterials has produced exciting results in the studies of cardiovascular disease models. A gene delivery hybrid complex composed of GO nanosheets and VEGF DNA plasmids has been shown to obviously increase the capillary density at the injection site and can be used to treat ischaemic heart disease [49]. The attachment of GO flakes to MSCs (MSC-GO) can significantly improve the therapeutic efficacy of angiogenesis and myocardial perfusion while avoiding the poor cell viability and limited therapeutic effect of using only MSCs [50] (Fig. 1). Thus, GO can act as both a scaffold for tissue engineering and a delivery system for gene therapy to achieve better therapeutic effects in the treatment of cardiac infarction. However, its long-term transformation and biological safety in vivo still require further study.

Tissue engineered vascular transplantation is an ideal vascular graft substitute when arterial stenosis, embolism, and rupture are difficult to repair. Nanofibres were found to be similar to collagen fibres in foetal and neonatal rat heart tissue under a microscope [51]. Moreover, the tensile properties of electrospun stents are closer to those of human arteries. Therefore, electrospun nanofibres have become commonly used nanosacffolds in vascular tissue engineering [52–54]. They can mimic the ECM of vascular tissue and can significantly promote endothelialisation, which is also the most effective strategy for thromboresistance. Many modifications have been investigated to control the diameter, direction and porous structure to obtain optimal cell compatibility. Three-dimensional poly-l-lactic acid nanofibrous scaffolds organized by electrospinning and hot embossing or soft lithography greatly improve the growth, proliferation and focal adhesion of endothelial cells [55, 56]. Porous elastic vascular grafts made of polycaprolactone nanofibre-reinforced poly (glycerol sebacate) have been shown to increase the infiltration of inflammatory M2 macrophages, thereby promoting angiogenic factor secretion and angiogenesis. The grafts also fused well with normal blood vessels in vivo, demonstrating that they are ideal in vivo vascular scaffolds [57]. In addition, a near physiological spiral nanofibrous tubular scaffold was demonstrated to improve the growth, distribution and function of human coronary artery endothelial cells by activating the mechanical pathway [58].

Electrospinning is a promising method for vascular grafts in early research, but no long-term in vivo studies have been reported, including the biomechanical properties after degradation, vascular patency, and neovascular tissue remodelling. It is necessary to fully describe this complete transformation process before clinical research to make this tissue engineering technology suitable for clinical application.

## Factors influencing the proangiogenic properties of nanomaterials

Inducing stem cells to differentiate into vascular-related cells and promoting cell adhesion, migration and proliferation play important roles in angiogenesis. A summary of the nanomaterials exhibiting angiogenic properties is provided in Table 2.

**Table 2:** The angiogenic property of nanomaterials in endothelial cells

Type of nanomaterials	Physicochemical properties	Result of related proangiogenic assay	The role of nanomaterial in angiogenesis
Inorganic nanomaterials			
Gold	Length 47 ± 0.4 nm, width 14 ± 0.2 nm [14] Hexagonal morphology, aspect ratio 1:1–1:1.5, length 30 nm [43] Spherical shape, 22 nm [44] Spherical shape, 7.6 ± 0.9 nm [38]	(1) Increase cell survival and proliferation of ECs (2) Increase vessel-like structures significantly (3) Increase expression of VEGF, ANG-1, and ANG-2	(1) Delivery system [14, 38] (2) Potent antioxidative effects [43] (3) As an optical switch of biological circuits [14, 44]
Cu_2_S	Nanoparticles, 200–600 nm [36]	Increase blood vascular networks and CD31 positive vessels	Controllable release of Cu ions [36]
HA	Nanoparticles, < 200 nm [20] Li doped into the HA, short acicular shapes, < 200 nm [28] nHA conjugated on the CHO functional groups of PLA scaffold [29] Nano-rod, micro-arc oxidation-H0.5, 223 nm [154] Embossed with nanoparticles, 75–250 nm [155]	(1) Improve the viability, adhesion and proliferation of ECs (2) Increase the expression of VEGF, CD31, HIF-1, vWF, VEGFR2, FGF, and ANG-1 (3) Accelerate the tube formation	(1) Delivery system [20, 28] (2) Promote the proliferation and adhesion of ECs at the initial stage [29, 33] (3) Immunomodulatory effects [154, 155]
TCP	Nanoparticles, 50 nm [21]	(1) Accelerate the proliferation of HUVECs (2) Enhance the secretion of VEGF and the gene expression of VEGF, VEGFR2 and HIF-1α	Promote cell adhesion and proliferation [21]
Bioactive glass nanoparticle/nanofiber	Sr doped bioactive glass nanofibers [17] Mesoporous spherical particles, < 300 nm, pore size < 7 nm [30] 440 nm, pore size 2–10 nm [31] Nanobioglass, ~ 30 nm [39]	(1) Improve the spreading and proliferation of HUVECs (2) More neo-blood vessel formation in CAM model (3) More newly formed blood vessels in vivo (4) Increase CD31 quantity and upregulation of VEGF expression	(1) Si ion release [30, 31, 39] (2) Delivery system [17]
Zinc oxide nanoflowers/nanoparticles	40–100 nm [111] 60 nm [112]	(1) Increase cell proliferation and DNA synthesis phase of HUVECs (2) Increase the migration of EA.hy926 cells (3) Increase the formation of vascular sprouting in the chick embryo angiogenesis assay (4) More blood vessels formation on the scaffolds in vivo subcutaneous implantation	The generation of ROS [110–112]
Terbium hydroxide rods/spheres	Tb^III^(OH)_3_, rod shape, diameter 111 ± 18 nm, length 847 ± 165 nm, nanospheres, 106 ± 19 nm [113]	Promote the recovery of intersegmental blood vessels pre-inhibited zebrafish	The generation of ROS [113]
Europium hydroxide nanorods/spheres	Eu^III^(OH)_3_, nanospheres, 21 ± 3, rod shape, diameter 36 ± 4 nm, length 215 ± 29 nm [113] EHN, nanorods, length ~ 150–200 nm, width ~ 40–50 nm [114]	(1) Increase cell viability of HUVECs and EA.hy926 cells (2) New blood vessel formation in chick embryo model (3) Higher tube formation assay of ECV-304 cells	The generation of ROS [113, 114]
Neodymium	Nanoparticles, nanocubes, nanorods, < 100 nm [98]	(1) Induce tube formation (2) Induction of angiogenesis in vivo CAM and chick aortic arch model assays (3) Activation of VEGF and VEGFR2 pathways	The generation of ROS [98]
GO	Monolayer thickness < 1 nm, width ~ 20 μm [27] PEI-GO[49] GO flakes, height ∼ 1.5 nm [50]	(1) Increase the adhesion, proliferation and migration of HUVECs (2) Form blood vessel like structures (3) The α-SMA, RECA-1, CD-31 positive cells	(1) Containing functional groups as delivery system [50] (2) Protein adsorption [27, 49] (3) M2 macrophage recruitment [27]
rGO	rGO: C/O ratio 8.6:1, 50 ng/mL, GO: C/O ratio 1.6:1, 10 ng/mL [59] Porous 3D structure, the ice segregation induced self-assembly technique, wall thickness 40–50 nm [48]	(1) Increase the proliferation of endothelial cells (EA.hy926) in vitro (2) Enhance angiogenesis and thickness of the blood vessels in CAM model (3) RECA-1 and laminin positive staining	(1) Induce a low level of ROS [59] (2) M2 macrophage recruitment [48]
TiO_2_	Highly ordered, vertically oriented TiO_2_ nanotubes, diameter 22–300 nm [80] TiO_2_ particles ~ 30–50 nm [81] Nanotubes, 90 nm [83]	(1) Increase the cell spreading and migration of primary human aortic endothelial cells (2) Decrease the proliferation and expression of collagen I and MMP-2 in primary human aortic smooth muscle cells	(1) Decrease expression of molecules involved in inflammation (2) Sense nanotopographical cues [80, 81, 83]
Cerium oxide nanoparticle	5–10 nm [116] Ce^3+^ concentration, 57%/27%, 3–5 nm [117]	(1) Promote viability and proliferation of HUVECs and ECV-304 (2) More blood vessel formation in chick embryo model	(1) Regulate oxygen concentration and activates HIF-1α (2) Reduce oxidative stress [116, 117]
Organic nanomaterials			
Nanofibrin	240 ± 5 nm [24]	Enhance tube formation in vitro	Promote cell adhesion and angiogenesis [24]
PLLA nanofibrous membrane	Porous PLLA electrospun membranes containing dimethyloxalylglycine loaded mesoporous silica nanoparticles [35]	Stimulate the proliferation, migration of HUVECs	(1) Nanotopology combines aligned electrospun fibers and nanopores can serve as a signaling mechanism to control cell growth and differentiation (2) Avoiding the detachment of nanoparticles (3) Delivery system [35]
Tetrahedral DNA	Triangular nanoparticles, formed by four ss-DNAs fragments [137]	(1) Promote the proliferation, migration and tube formation of ECs (2) Increase the expression of VEGFA, VEGFR2	Low biotoxicity, nuclease resistance, relative stability and programmability [137]

*HA* hydroxyapatite, *TCP* tricalcium phosphates, *GO* graphene oxide, *rGO* reduced graphene oxide, *PLLA* Poly-l-Lactide, *EC* endothelial cells, *HUVEC* human umbilical vein endothelial cell

**Fig. 2:**
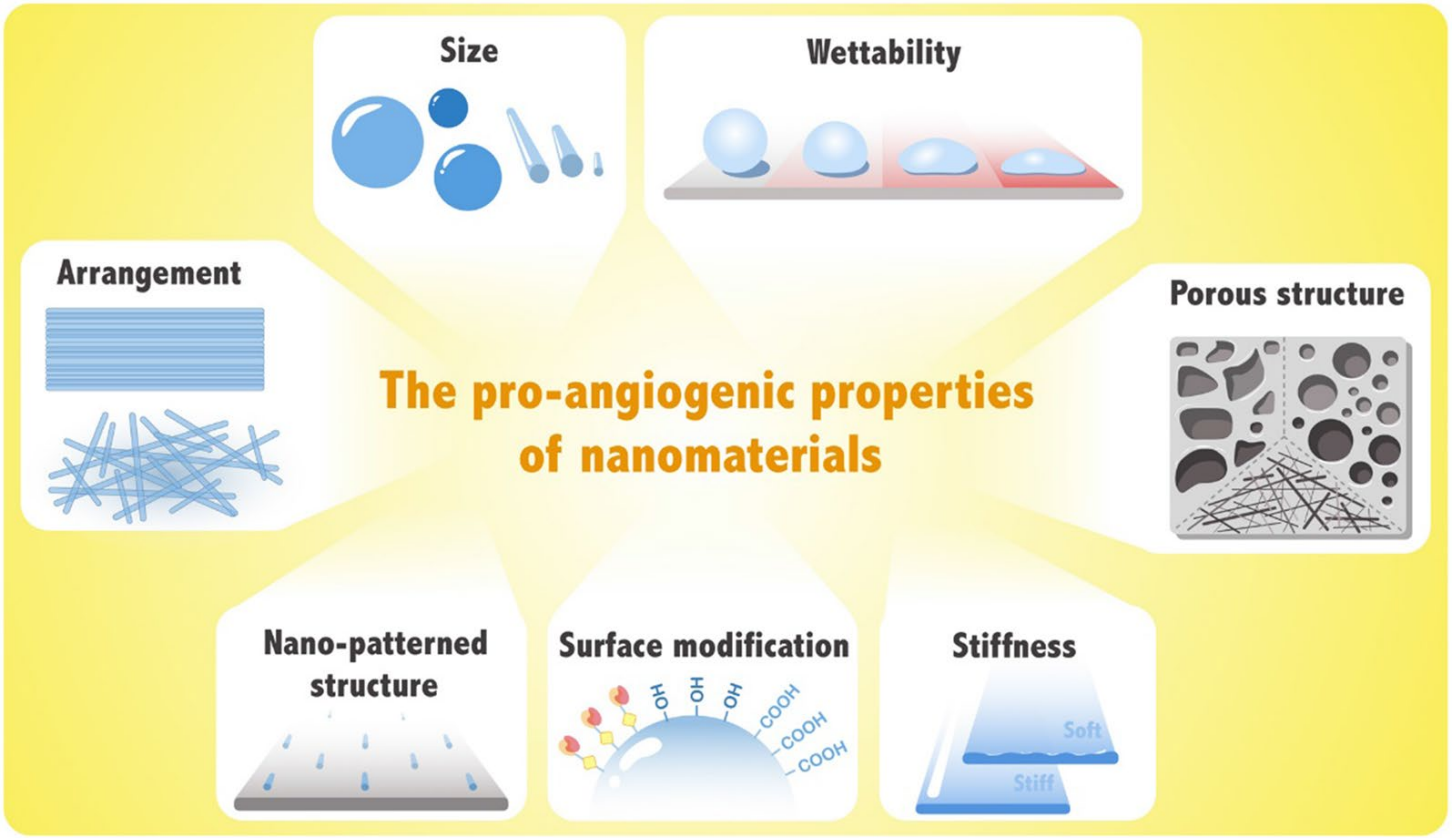
Schematic illustration of factors influencing the angiogenic properties of nanomaterials

Generally, the proliferative capacity, adhesion and diffusion capacities of endothelial cells on the surface of nanomaterials are greater than those on planar surfaces, possibly due to the changes in the physicochemical characteristics [59, 60]. In detail, the characteristics of a nanomaterial influence its angiogenic properties (Fig. 2).

### Surface chemical modifications

Nanomaterial surface modification is one method for modifying biological materials. The morphology, chemical properties and wettability of the substrate surface change accordingly, thus affecting cell activity and biocompatibility.

Functional peptide-coated gold nanoparticles promote endothelial cell capillary formation based on the proangiogenic function of peptides [38, 61, 62]. Carbon nanoparticle-grafted functional groups containing oxygen and nitrogen (e.g., amines and amide groups) reverse the negative zeta potential of unmodified carbon nanotubes, and chitosan-mediated cation electrodeposition-coated nanoparticles interact better with the negatively charged cell membrane, thereby increasing the adhesion, activity and proliferation of HUVECs and vascular smooth muscle cells [63, 64]. Khor et al. compared the characteristics of carboxylic acid/poly(ethylene glycol) methyl ether/methyl ester/tertiary amine ester-modified polymer nanoparticles, and the results suggested that tertiary amine ester-functionalized nanoparticles have a stronger cell binding capacity in static and simulated intravascular fluids than other modifications [65], possibly due to the adsorption of different proteins or the size of the nanoparticles with different surface modifications [66]. However, the binding force between tertiary amine ester-modified nanoparticles and cells resists the influence of haemodynamic separation, although larger size increases the drag force of the fluidic flow [65]. However, amine-terminated dendrimer-modified silica nanoparticles exhibit haematotoxicity because of positively charge activate fibrinogen and plasminogen simultaneously [67]. The nanoparticles in scaffold may enter the cells or blood flow accidentally, so the biological interactions and safety should not be ignored either.

Miller et al. asserted that a nanostructured poly (lactic-co-glycolic acid) film treated with sodium hydroxide is not conducive to the function and activity of endothelial cells because of surface chemical effects [68]. They obtained a nanostructured surface using a casting technique, which increased the density of endothelial cells while eliminating surface chemical effects.

Nanoscaffolds immobilized with growth factors exhibit enhanced stability and biological effect. VEGF and angiopoietin 1 encapsulated by nano-sustained release polylactic acid microspheres enhance the proliferation and differentiation of adipose MSCs into endothelial cells, which is conducive to angiogenesis [69]. A nanofibre scaffold loaded with fibroblast growth factor (FGF) or VEGF compared to one without growth factors has been demonstrated to significantly promote angiogenesis and inhibit thrombosis [70–72]. The functionalization of biomolecules (such as heparin) on nanofibre scaffolds increases angiogenesis at the implant site without the addition of exogenous growth factors [73].

### Stiffness

Stiffness, as an important mechanical feature of the matrix, can affect cell morphology, proliferation, migration and differentiation. Studies have shown that the adhesion, proliferation and expression of the proangiogenic-related factors of endothelial cells increase with substrate stiffness [74]. With the increase in substrate stiffness, endothelial cells migrate farther and deposit more linearly and aligned on fibronectin fibres [75, 76]. A stiff polydimethylsiloxane substrate (E = 195 kPa) has been shown to be more conducive to angiogenesis and the differentiation of adipose-derived MSCs than a soft substrate (E = 15 kPa) [77]. The stiffness of the substrate not only affects cell differentiation and movement but also regulates the uptake of nanoparticles. The bovine aortic endothelial cell membrane expanded, and actin fibre formation increased on a stiffer substrate (E = 5.71 ± 0.51 kPa), resulting in a higher nanoparticle uptake [78]. These results indicate that the stiffness of nanoscaffolds may also play a role in the regulation of angiogenesis. However, nanofibres obtained via acid-assisted treatment are softer (the compressive modulus was approximately 6 kPa) than those obtained by salt leaching, which promotes the differentiation of bone marrow MSCs into endothelial cells and in vivo vascularization [79]. Whether this is due to the difference in the elastic modulus or chemical treatment is up for debate.

### Geometric shape

The growth, proliferation, and migration of vascular cells are regulated by the structure, orientation, and porosity of nanomaterials.

#### Arrangement

Compared with amorphous or randomly arranged nanofibres or nanotubes, the directional alignment benefits the growth of vascular smooth muscle cells and endothelial cells [80–83]. Studies have demonstrated that the interaction between oriented nanostructures and cells promotes cell alignment and directional growth by reassembling the actin cytoskeleton while inhibiting inflammation, thus maintaining intact intercellular junctions [84, 85].

A titanium dioxide nano-/micropattern on the surface of titanium fabricated by photolithography and anodic oxidation technology is conducive to cell growth along the grooves of the surface. The proliferation and differentiation of MSCs into vascular smooth muscle cells has been shown to be more effective on nano-/micropatterned titanium dioxide surfaces than on single titanium nanotubes or flat surfaces [86].

#### Dimensions

**Fig. 3:**
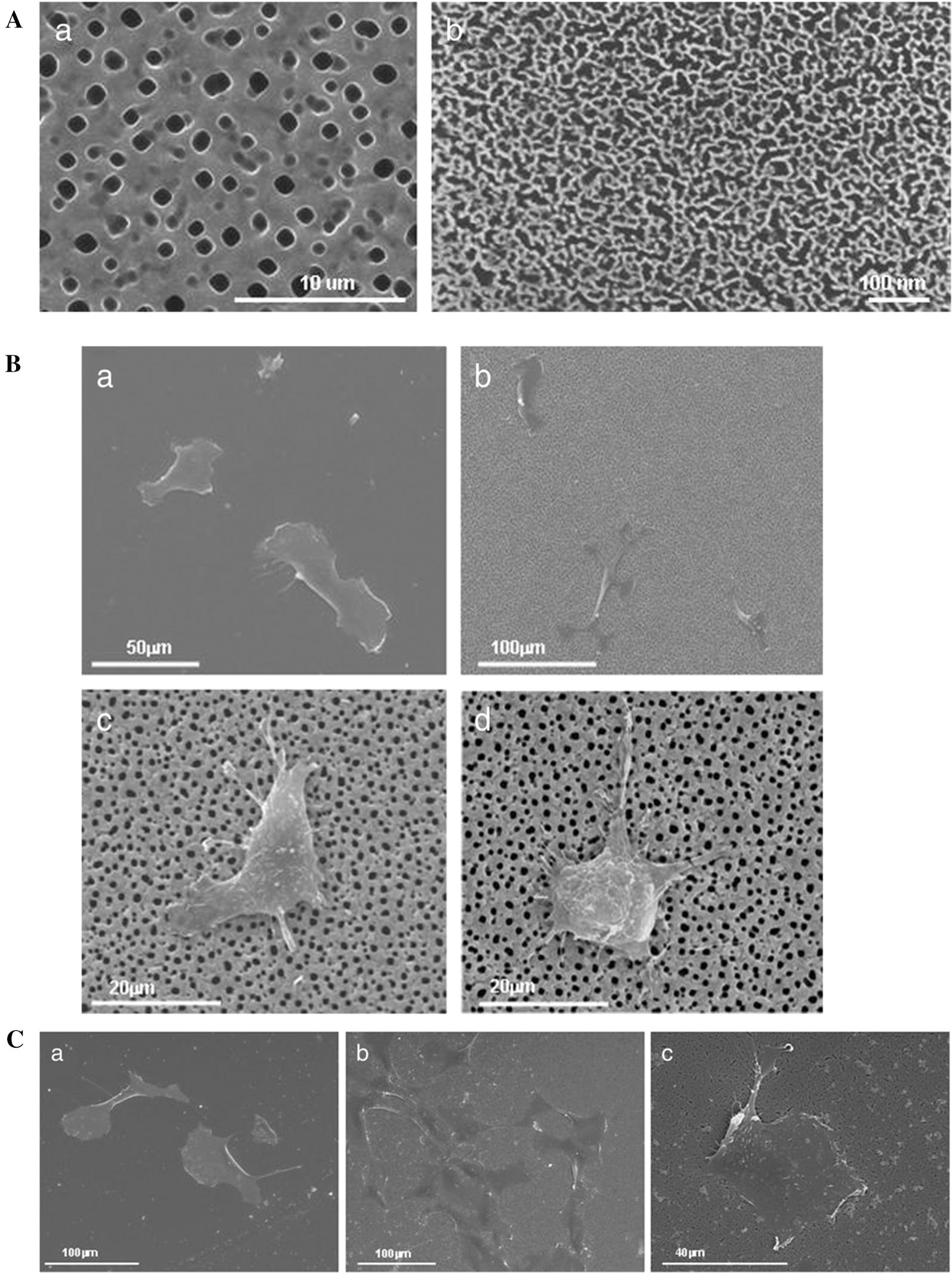
Human aortic endothelial cells on the porous silicon substrates. **A** Morphological characterization of macroporous (a) and nanoporous (b) silicon substrates. **B** SEM characterization of endothelial cells on macroporous silicon. **C** SEM characterization of endothelial cells on nanoporous silicon. Reprinted with permission from [95] Copyright 2014 Springer

Nanopatterned materials affect fibronectin absorption, the formation of focal adhesion and Rho-A GTPase and collagen expression, thus affecting the growth and spread of cells [87]. Four endothelial cell types, i.e., HUVECs, human dermal microvascular endothelial cells, human saphenous vein endothelial cells and HAECs, exhibit oriented and aligned growth on anisotropic ordered nanopatterns. However, only the proliferation of HUVECs is significantly reduced on both 400 nm and 800 nm pitches. Cell migration was found to increase in higher topographic features from 400 to 4000 nm, with the exception of HAECs [88]. HAECs cultured on scaffold surfaces with titanium dioxide nanotubes with diameters of 30 and 100 nm are also not significantly different [80], which suggests that heterogeneous spreading and angiogenesis functions are involved in different endothelial cell types. Poly (styrene) and poly (4-bromostyrene) consisting of nanohills 13 and 35 nm in height presented greater adhesion and better spreading of HUVECs and human microendothelial cells. The best endothelialized poly (styrene) and poly (4-bromostyrene) surfaces of 13 nm nanohills exhibited the lowest monocyte and granulocyte adherence [89, 90], and the oriented nanotopography surface and the lower depth structure (< 40 nm) always exhibited fewer platelets adhesion [91, 92]. A comparison of gradient nanopatterned plates consisting of nanopillars with different diameters ranging from 120–200, 200–280, to 280–360 nm has shown that the cytoskeletal integrity and focal adhesion of human endothelial colony-forming cells (hECFCs) on nanopatterned plates are better than the cytoskeletal integrity and focal adhesion of hECFCs on flat plates, and filamentous outgrowth increased significantly in the range from 120 to 200 nm [93]. The pore size of nanomaterials also influences the morphology and adhesion of human endothelial cells. HAECs adhere to nanoporous silicon and diffuse on the surface of the material with multiple thin filopodia, which are pseudopodia that protrude from the cell membrane into the macroporous matrix [94, 95] (Fig. 3). These results might provide clues for the design of nanoengineered implants regulating the growth rate of angiogenesis.

The size and shape of nanoparticles have become important considerations in the interaction with endothelial cells and angiogenic effects. Usually smaller nanoparticles lead to increased intracellular endocytosis and reactive oxygen species (ROS) production, which causes DNA damage and cell apoptosis [96]. However, it is also closely related to the characteristics of the material itself. Cerium oxide nanoparticles can exist in both trivalent and tetravalent state, and the oxygen vacancy on the surface can eliminate the effect of oxidative stress, which has become a rare earth metal oxide of great concern in the biomedical field [97]. It has been found that cerium oxide nanoparticles has shown potential for tube formation at only a size of < 15 nm, possibly due to the increase in the size of nanoparticles and the decrease in the catalytic active surface area for the oxygen modulation pathway. Neodymium nanoparticles also play a role in promoting angiogenesis via shape-dependence. Experiments in vivo and in vitro show that spherical neodymium has the best biocompatibility to promote cell proliferation but exhibits the poorest redox-modulating effect. An evaluation of smooth muscle cells, endothelial cells and pericytes showed that rod-shaped nanopolymorphs of neodymium had the best angiogenic effect [98]. Comparing to the hydroxyapatite nanoneedles and hydroxyapatite nanoflakes, the hydroxyapatite nanospheres showed greater angiogenic potential probably due to the cellular uptake and autophagy activation caused by nanoparticle morphologies [99].

A nanoparticle of the proper morphology balances blood flow resistance and optimal adhesion, which is important for the targeted therapy as delivery vector design. In general, it is believed that the binding of nanosized particles to endothelial cells is lower than that of microparticles in the blood and circulation, while the binding of disc- or rod-shaped particles is higher than that of spherical particles [100]. The aspect ratio can also be used as an optimized factor for biological nanoparticles. Tobacco mosaic virus nanorod particles at different aspect ratios mediate endocytosis through different pathways. The uptake of short tobacco mosaic virus rods with aspect ratios of 4 and 8 was mainly mediated via clathrin in HUVECs, while tobacco mosaic virus rods with an aspect ratio of 17 were mainly mediated by caveolae and microtubules, which led to faster cellular uptake [101].

### Wettability

The wettability of the biomaterial surface can change the interaction of the material with the surrounding cells and affect the adhesion and differentiation of the surrounding matrix, proteins, growth factors and cells. Wettability is characterized by hydrophilic and hydrophobic properties, and the regulation of wettability depends on the material and cell type [102]. Polymers with higher wettability can better adsorb serum and/or cell protein molecules and promote the growth of human endothelial cells [103]. HUVECs grew better on hydrophilic dental implant surfaces than on smooth hydrophobic surfaces [104]. Endothelial progenitor cells can produce more angiogenic factors, including VEGF-A, endothelial nitric oxide synthase (eNOS) and inducible nitric oxide synthase (iNOS), on hydrophilic rough surfaces, although their adhesion and proliferation are poor [105]. Most studies have indicated that the nanoscale morphology and roughness of a scaffold can increase the surface texture, surface energy and wettability. Therefore, it is speculated that the difference in wettability caused by various nanomaterials and topologic features is one aspect affecting the biological process of angiogenesis.

## Effects and underlying mechanisms of nanomaterials in different stages of angiogenesis

**Fig. 4:**
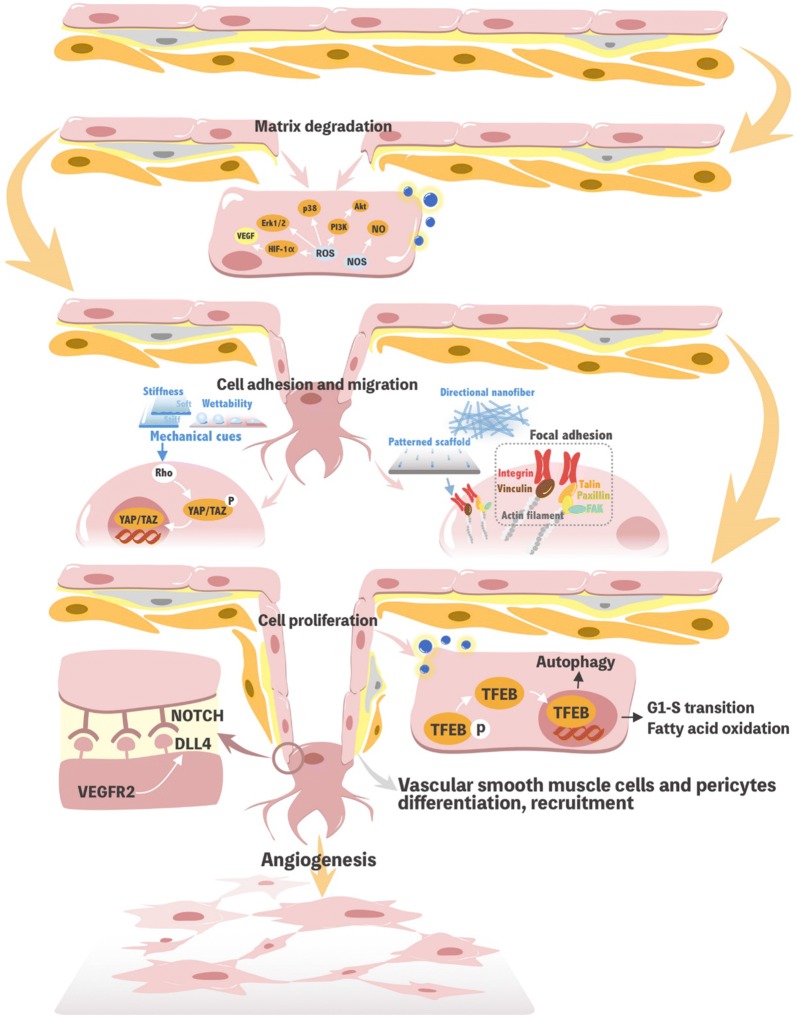
Illustration of possible mechanisms of nanomaterials in angiogenesis

Angiogenesis is a dynamic and complex process that begins with endothelial cells. Stimulated by proangiogenic factors, endothelial cells migrate directionally, followed by adjacent cell proliferation, ultimately forming tubular structures. The following section will introduce how nanomaterials affect the cell behaviour and play a role in the signalling mechanisms that promote angiogenesis (Fig. 4).

### Promoting endothelial cell migration

Under the guidance of microenvironment signals (such as VEGF), quiescent endothelial cells in the linings of vessel walls activate and differentiate into endothelial tip cells, which possess elevated expression levels of Dll4 and vascular endothelial growth factor receptor (VEGFR) 2. Endothelial tip cells initiate sprouting and migration, which is one of the early hallmarks of angiogenesis [106]. The molecular mechanism of nanomaterials participates in this biological process induced by VEGF through the following aspects.

#### Activation of redox signalling

Inducing the activity of matrix proteases is one of the initial characteristics of angiogenesis and can provide interstitial space for endothelial cell migration. ROS (especially superoxide anion, O_2_^• −^ and hydrogen peroxide, H_2_O_2_) and/or reactive nitrogen species (RNS) are redox signalling molecules that play important roles in regulating various cell signalling pathways and biological effects [107]. Studies have shown that a proper ROS concentration can increase the expression levels of the transcription factors NF-kappaB, activator protein 1 and E26 transformation specific 1 in endothelial cells and can bind to the promoters of matrix metalloproteinases such as stromelysin, collagenase, and urine plasminogen activator [108]. ROS is not simply a cytotoxic factor but is also an important regulator of cell physiological function. Additionally, it can activates HIF-1α, promoting the release of other angiogenic factors, such as VEGF [107]. Nitric oxide (NO) is the main RNS produced by cells. As a downstream effector of VEGF, eNOS generates NO, which can increase the expression of matrix metalloprotein 13, destroy collagen and activate the PI3K-Akt signalling pathway, leading to the migration of endothelial cells [109].

Nanomaterials, such as nano zinc oxide [110–112], lanthanide nanoparticles [113], and europium hydroxide [114], promote the migration and early tube formation of HUVECs. These nanomaterials activate kinases such as Akt, extracellular regulated protein kinases (ERK) 1/2, mitogen-activated protein kinase (MAPK) p38 [115] and eNOS, which are ROS-dependent, even though the production of ROS is always associated with cytotoxicity. Nethi et al. designed functionalized nanoconjugates of (6-{2-[2-(2-methoxy-ethoxy)-ethoxy]-ethoxy}-hexyl) triethoxysilane and samarium doped cerium oxide nanoparticles (MTS-SmCeO_2_) to promote endothelial cell viability and blood vessel formation in a chick embryo model by decreasing high levels of ROS in EA.hy926 cells to optimal levels and enhancing the activation of the p38 MAPK/HIF-1α signalling pathway [116]. Cerium oxide nanoparticles also utilize oxygen vacancies on the surface of the lattice to scavenge free radicals and reduce the damage caused by oxidative stress, thus stabilizing HIF-1α and leading to angiogenesis [42, 117]. This may be related to the different basic levels of ROS in different cells. The bidirectional regulation of ROS by nanomaterials can be used as a novel strategy to promote angiogenesis for medical applications and can undoubtedly achieve good results.

#### Regulating cytoskeleton rearrangement

VEGF activates the ERK and Akt signalling pathways, leading to metalloproteinase secretion, which degrades the basement membrane. This activation and subsequent degradation are followed by cytoskeletal remodelling with F-actin polymerization and filopodia extension, allowing endothelial cells to migrate into the wound area [118].

**Fig. 5:**
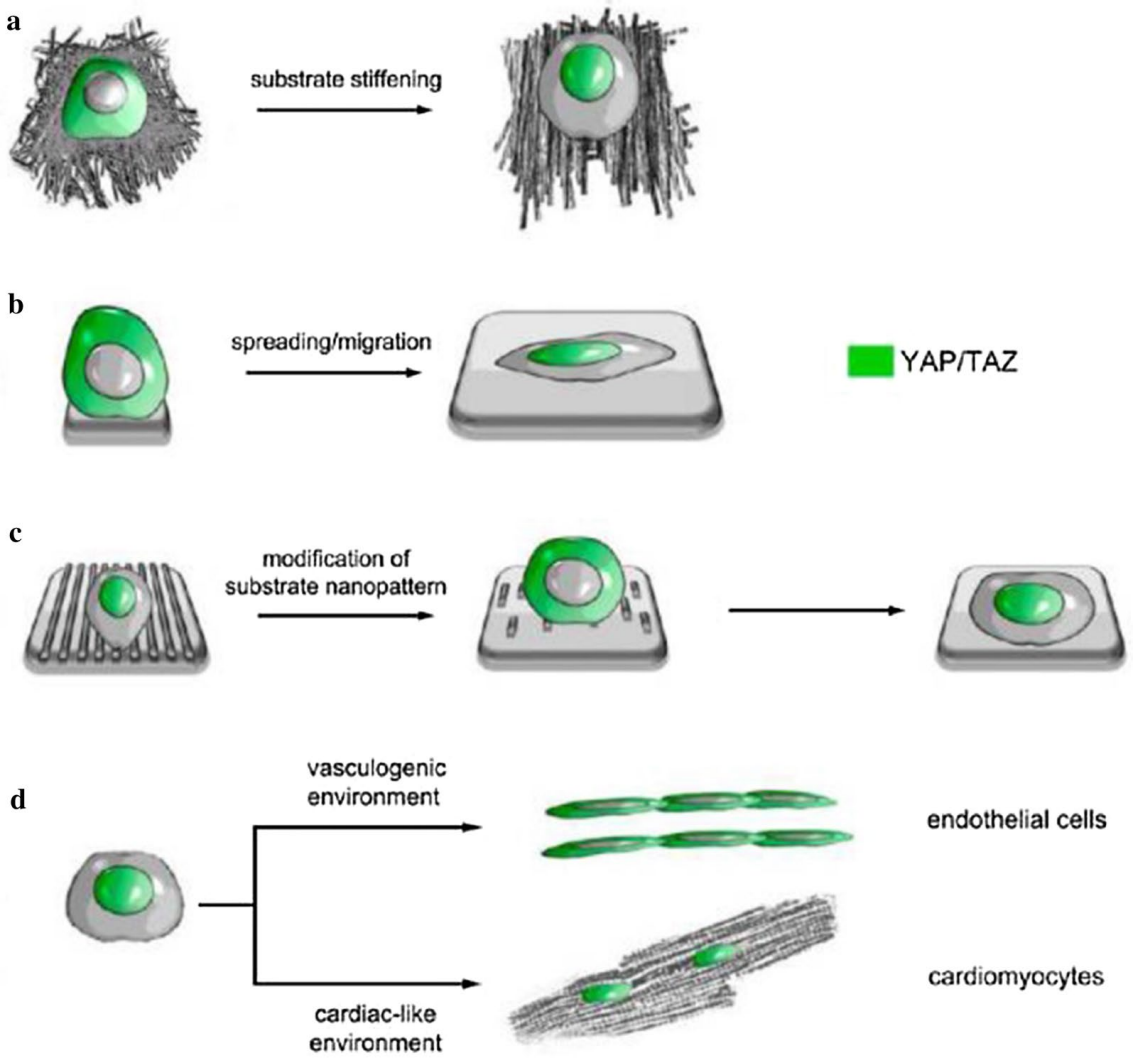
YAP/TAZ control cardiac progenitor cell fate by acting as sensors of extracellular matrix composition. YAP/TAZ activity as transcriptional coactivators is regulated via their phosphorylation in the cytoplasm. Phosphorylated YAP/TAZ are thought to be inactive when retained in the cytoplasm. Nuclear shuttling is triggered in cardiac progenitor cells by substrate stiffening (**a**), cell spreading or migration (**b**), and modifications in substrate nanopattern (**c**). More importantly, the regulation of YAP/TAZ intracellular localization is required for cardiac progenitor cell fate decision (**d**). Reprinted with permission from [121]. Copyright 2014 American Chemical Society

Small Rho GTPase mediates endothelial cell cytoskeleton arrangement and tension, which are involved in blood vessel development. ECM stiffness regulates VEGFR2 expression in human dermal microvascular endothelial cells and HUVECs by controlling p190Rho GTP, GATA binding protein 2 and TFII-I, thereby compensating for the instability of soluble growth factors and synergistically contributing to endothelial cell migration and vascular network formation [119]. Human dermal microvascular endothelial cells and microendothelial cells cultured on matrices of different hardness levels can exhibit differences in yes-associated protein (YAP) and transcriptional coactivator with PDZ-binding motif (TAZ) phosphorylation, which occurs in response to Rho GTPase activity and actin cytoskeleton tension, thereby affecting cell migration and proliferation [120]. YAP/TAZ acts as a mechanical sensor in the cell that is activated by ECM rigidity, which is necessary for the differentiation of MSCs induced by ECM and the survival of endothelial cells in a geometric pattern [121] (Fig. 5).

Nanomaterials not only imitate the ECM but also change the surface pattern, hardness and elasticity of the scaffolds. Cui et al. reported F-actin assembly and increased filopodia in hECFCs on gradient nanopattern plates through the activation of Rho-associated protein kinase signalling; hECFCs on flat plates did not express these proteins [93]. The nanostructure and substrate rigidity determine the adhesion, spreading, differentiation and tube formation of cardiac progenitor cells by changing YAP/TAZ expression [121]. Nevertheless, details regarding the cellular mechanotransduction mediated by nanomaterials during angiogenesis have not been reported. These molecular mechanisms may regulate cytoskeletal arrangement and cell tension, thus playing roles in different steps of angiogenesis in response to the physical signals of nanomaterials and nano-topographic features, but this speculation requires further investigation.

#### Focal adhesion formation

Integrin is composed of α and β subunits through noncovalent bonding. It mediates focal adhesion and then increases the adhesion and migration of HUVECs, which is the primary adhesion mechanism between cells and the ECM. Integrin combined with actin filaments, vinculin, talin, focal adhesion kinase (FAK) and paxillin lead to focal adhesion formation, which is crucial for the modulation of mechanosensing [122, 123]. The activation of FAK by clustered integrins linked to the cytoskeleton regulates the adhesion and migration of vascular endothelial cells through signalling pathways such as FAK-Rho GTPase and FAK-PI3K. The phosphorylation of paxillin Ser85, which is bound to the talin C-terminal junction domain, regulates focal adhesion formation and cell migration [124].

**Fig. 6:**
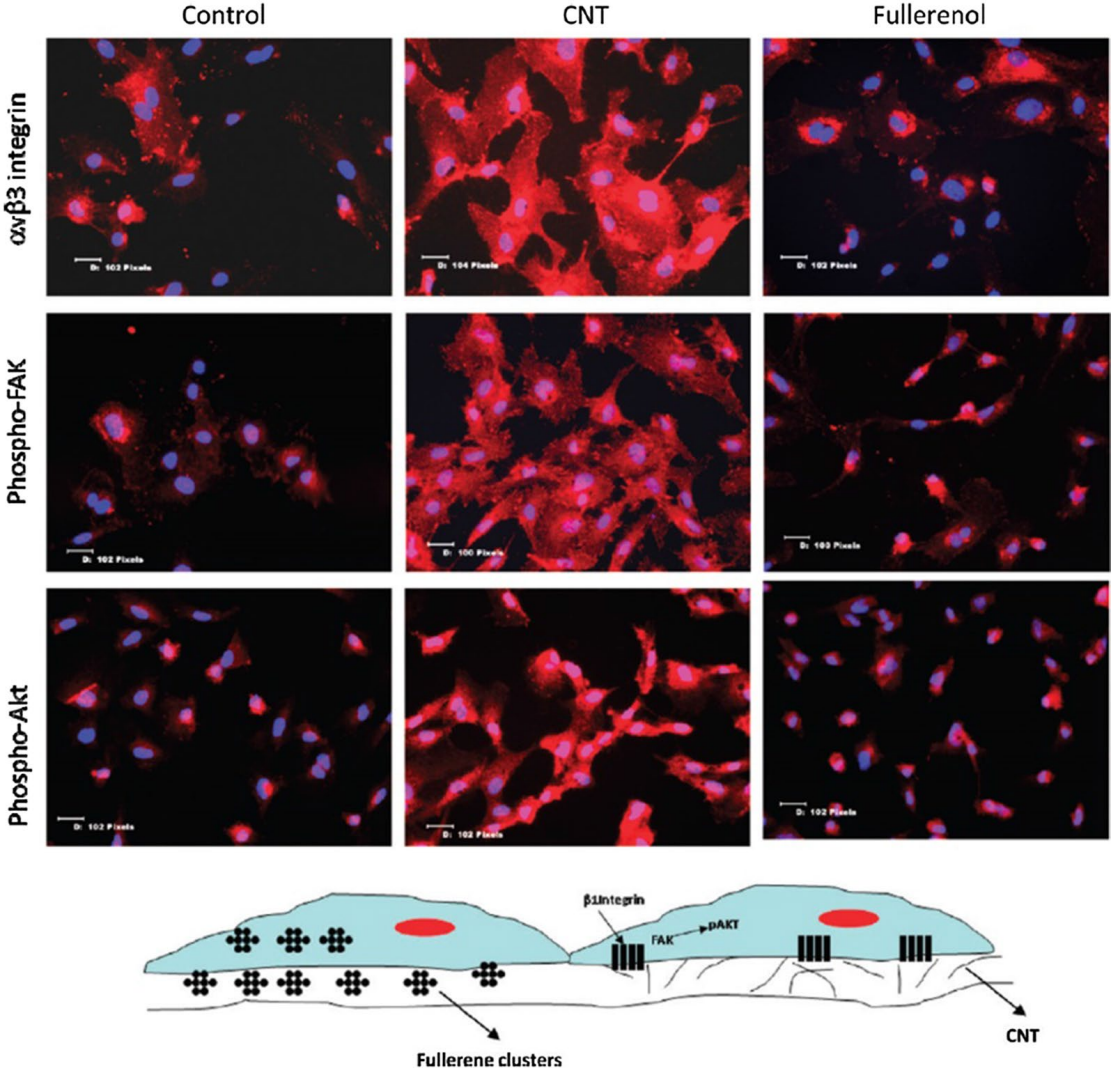
Effect of HUVECs treated with the single-walled carbon nanotubes (CNT) and fullerenol. Cartoon shows the mechanism through which the CNTs likely promote angiogenesis. The clustering of integrins results in phosphorylation of FAK, which can then activate PI3K that phosphorylates Akt, which has been implicated in angiogenesis. Reprinted with permission from [127]. Copyright 2010 American Chemical Society

Integrin-β1 expression in endothelial cells was increased on nanofibrous fibroin scaffolds compared to those cultured on microfibrous fibroin. Compared with non-patterned scaffolds, scaffolds with oriented nanofibres activate integrin α1, which promotes the directional growth and spread of endothelial cells [82, 125]. The zirconium nanostructure formed on Ti-6Al-4V has lower hydrophilicity and protein adsorption than the micro-/nano-zirconium oxide alloy layer, but the cellular adhesion and activity of cells are significantly higher, possibly due to a lack of clustered integrins on the micro-/nanostructure, resulting in the inability to form focal adhesions [126]. Carbon nanotubes have been shown to upregulate αvβ3 integrins in endothelial cells and to activate focal adhesions as well as downstream PI3K-Akt signaling [127] (Fig. 6). However, fullerenols exert the opposite effects. Therefore, not only the nano-topological structure but also the different nanomaterials and their physicochemical properties play a complex regulatory role in the biological activity of endothelial cells.

Bioactive protein and the adhesion density are key regulators on the surfaces of nanomaterials. The bioactive RGDS loop and PHSRN sequence within a 3.2 nm distance could simultaneously bind the α5β1 integrin, whereas a larger distance between these two peptide sequences reduces the capacity to activate integrin. Therefore, constructing nanofibre scaffolds with the correct synergy and spacing of cyclic RGDS and PHSRN epitopes would support the spreading of HUVECs cells by upregulating α5β1 integrin [128]. An RGD spacing of 44 nm on the nanoscale surface of silicon is most conducive to cell spreading [129]. When the spacing between the protuberance of a nanostructured surface is greater than 70 nm, the influence of integrins is eliminated, and focal adhesion formation and cell diffusion are inhibited [130]. These parameters are of great significance for the future design of biomaterials and the control of tissue biological activity.

#### Transition to glycolysis

Cytoskeletal remodelling and the migration of endothelial tip cells depend on adenosine triphosphate derived from glycolysis. Phosphofructokinase-2/fructose-2, 6-bis- phosphatase 3 and hexokinase 2 are important activators of glycolysis, and VEGF and HIF-1α can increase phosphofructokinase-2/fructose-2, 6-bis-phosphatase 3 expression. FGF stimulation elevates hexokinase 2 levels, leading to pseudopodia formation and cellular migration [131–133].

It has been found that HEK293 is more easily induced than other tumour cells at the nontoxic concentrations of nano-silver, resulting in a transition from aerobic metabolism to anaerobic glycolysis. Meanwhile, smaller nanospheres are more likely to cause a metabolic shift than larger nanospheres or nanoplates [134]. Whether they participate in the biological effects of endothelial cells through this pathway remains unknown. The changes in endothelial cell metabolism induced by nanomaterials can provide a new perspective on how to promote angiogenesis.

### Promoting endothelial cell proliferation

Triggered by angiogenic factors, quiescent endothelial cells exhibit stalk cell phenotypes with ID1, ID2, HES1 and FLT1, which contribute to the proliferation and help lengthen the sprouting vessels, leading to the formation of a new lumen [135]. In addition to the MAPK and PI3K/Akt signalling pathways, Notch signalling and autophagy are also emerging mechanisms by which nanomaterials participate in endothelial cell proliferation.

#### Notch signalling

The migration and proliferation of endothelial cells are not completely independent. The activation of an individual cell’s migration is always accompanied by the activation of adjacent cells. VEGF activates the expression of Dll4 in tip cells, followed by inducing Notch transcription factors and inhibiting VEGFR2 signalling in neighbouring stalk cells. The VEGF-Dll4/Notch feedback system drives the dynamic phenotype of the tip and stalk cells in the growing vessels, leading to the formation and maturation of the functional vascular plexus [136]. Tetrahedral DNA nanostructures are novel and biocompatible nanomaterials that promote the angiogenesis of endothelial cells by upregulating Notch signalling [137]. However, the exact mechanism underlying these effects has not been explored.

Stalk cells synthesize nucleotides, proteins and/or lipids to support cell division and proliferation [136]. Therefore, cellular metabolism differs between tip and stalk cells. Studies have found that Notch signalling in endothelial cells is also influenced by plasma glucose levels or fatty acids utilization to promote angiogenesis [136, 138]. At present, many studies on the metabolic changes induced by nanomaterials have focused on cytotoxicology. We speculate that nanomaterials may also induce stalk cell behaviour and corresponding biological responses by glutamine metabolism or the breakdown of fatty acids, but the concrete mechanism is still unclear.

#### Inducing autophagy

Autophagy is a biological process by which autophagosomes are formed and then fused with lysosomes to degrade autophagic contents by lysosomal protease. Autophagy can promote angiogenesis under inflammatory stimulation, hypoxia or high-glucose microenvironments [139, 140].

Transcription factor EB (TFEB) is a master regulator of autophagy and lysosomal biogenesis. TFEB promotes endothelial cell proliferation by activating the autophagic flux and regulating the G1-S transition, which is conducive to angiogenesis [141, 142]. In addition, the metabolites of autophagic lysosomes can be recycled into amino acids and lipids to produce adenosine triphosphate [143], which is also one of the mechanisms of endothelial cell proliferation mentioned above. In the local microenvironment, the protective effect of autophagy is also conducive to the proliferation and differentiation of endothelial progenitor cells into endothelial cells [144].

Most studies have reported that the autophagy induced by these nanomaterials is an important mechanism of cytotoxicity. In some cases, nanomaterials may induce protective autophagy, which promotes cell survival. For example, silver nanoparticles caused the nuclear translocation of TFEB, enhancing autophagy and cell survival [145]. Whether this is one of the mechanisms of promoting angiogenesis induced by silver nanoparticles is unknown. Targeted rapamycin micelle nanoparticles have been shown to play a protective role in the vascular endothelium under oxidative stress by inhibiting the release of pro-inflammatory cytokines [146]. Thus, autophagosome fusion inhibited NACHT, LRR and PYD domain-containing protein 3 (NLRP3) inflammasomes and reduced the secretion of interleukin (IL) 1β, which might be involved in the proangiogenic effect of multi-walled carbon nanotubes [147].

### Recruitment of mural cells

The ECM of vascular tissue is nanostructured; thus, nanomaterials are very valuable in the construction of biomimetic vascular tissue scaffolds. Endothelial cells on nanomaterials contribute to recruiting pericytes and vascular smooth muscle cell to form stabilized and functional vessels. The addition of nanomaterials has been found to significantly increase the growth and proliferation of smooth muscle cells and to decrease platelet adhesion [148]. The adhesion, diffusion and proliferation of vascular smooth muscle cells are significantly enhanced on polycaprolactone nanowires [149] and poly-l-lactic acid nanofibre scaffolds [52]. It has been reported that titanium dioxide nanoparticles coated on Ti-6Al-4 V significantly increased the adhesion, diffusion and proliferation of human aortic smooth muscle cells [81]. However, titanium dioxide nanotubes [150] have a negative effect on the proliferation of vascular smooth muscle cells, which may be due to various characteristics of the nanomaterials, including the type, size and surface charge [151].

Bone marrow MSCs can not only differentiate into endothelial cells and vascular smooth muscle cells but also secrete growth factors to participate in the angiogenesis process. The ECM protein-adsorbed GO flakes could improve the survival and adhesion of MSCs [50]. The titanium dioxide micro/nano interface also promotes MSC adhesion, proliferation and differentiation into vascular smooth muscle cells and endothelial cells [86], thereby promoting angiogenesis.

The implicated signalling pathways in mural cell differentiation, recruitment, or their attachment to endothelial cells vary in different tissues [152], and enhancing the integrity of the endothelial barrier is also essential for reparative angiogenesis. However, studies on the effects of nanomaterials on pericytes and smooth muscle cells are relatively rare, and little attention has been paid to the interactions between endothelial cells and mural cells; moreover, their coordination with nanomaterials has not been studied. Therefore, employing the synergistic effects of nanomaterials themselves to promote cell spreading, differentiation and stable barrier formation as well as to avoid lumen stenosis and thrombosis would come into the focus of angiogenesis.

## Limitations and prospects

### Effects of the microenvironment

Studies evaluating nanomaterial implantation have focused mostly on the phenomenon of blood vessel formation but have neglected the complementary roles of the local microenvironment and adjacent tissues.

Tissue damage or scaffold implantation can induce local or systemic inflammation. Macrophages are the main types of immune cells involved in the inflammatory response and participate in the balance of tissue damage and repair. They can be activated into M1 (pro-inflammatory) and M2 (pro-healing) macrophages in different microenvironments. Biomaterials, especially nanostructured materials, can regulate biological functions by stimulating the polarization of M2 macrophages, which is a promising way to obtain favourable tissue repair and regeneration [153]. Nano- hydroxyapatite has been reported to increase the expression levels of M2 markers [154, 155] and to facilitate angiogenesis in vitro. However, research has only shown the involvement of hydroxyapatite in bone regeneration, and its regulatory factors and mechanisms have not been reported in detail. Moreover, notably, M1 macrophages can also promote sprouting via the secretion of VEGF, FGF, IL-8, chemokine ligand 5, and tumour necrosis factor α, and cytokines also play a role in angiogenesis by upregulating glycolysis. It has been suggested that M1 and M2 macrophages are involved in the process of angiogenesis from germination to maturation rather than in a substitution relationship [156]. Therefore, properly designed nanomaterials regulating the M1–M2 macrophage balance avoid the adverse effects of excessive inflammation on tissue healing and remodelling and promote early vascularization, which will be the focus of future research.

In response to different microenvironments, nanomaterials selectively exerting protective biological effects between normal and diseased cells are of increasing importance. It is known that angiogenesis is not conducive to the treatment of tumours. A redox modulator fabricated by nano-biotechnological intervention exhibits different bioactivity effects under normal conditions or those mimicking tumour angiogenic conditions. In the physiological settings, the modulator activates the VEGFR2/p42 MAPK signalling pathway, which helps the cell survival and tube formation in vitro and in vivo through the release of ROS at moderate levels. However, the modulator hinders the angiogenic process under a VEGF-stimulated condition, mimicking the tumour microenvironment [157]. This finding will provide new inspirations for further exploring the application of nanomaterials in promoting functional vascularization in microenvironment in vivo. How to control the biological response between nanomaterials and endothelial cells better in other complex and pathological conditions remains a noteworthy challenge for future work. Furthermore, a biological model closer to the microenvironment in vivo, such as fluid stress and hypoxia, should be included in vitro study to present the biological effects of nanomaterials more clearly and rationally.

### Diversification of nanomaterials

In the literature review, it was found that the nanomaterials used in each study vary greatly, yielding different results in biosafety assessments and gene expression profiles. For example, gold nanoparticles can promote local angiogenesis of injured skin in vivo [44], but can reduce blood vessel formation in the chick chorioallantoic membrane model [158]. Although the size of nanoparticles used in the two studies is similar, the concentration of gold nanoparticles used in the animal model is not shown. Zinc oxide nanoparticles exhibited toxicity in HUVECs due to the release of zinc ions [159], but Barui et al. found that zinc oxide nanoflowers can promote the proliferation and migration of HUVECs and enhance angiogenesis in the chick embryo model [110]. However, the more detailed physicochemical properties of zinc oxide nanoflowers, such as the content of zinc ions and nanostructure stability, were never displayed. The inconsistent effect may be due to the different nanotechnology, dosages and other parameters of nanomaterials. It is well known that the characteristic of nanomaterials have an important effect on the biological response of endothelial cells. However, the description of physicochemical properties of nanomaterials in studies is relatively imperfect, such as residues in the synthetic process of nanomaterials, the dispersion and size distribution of nanoparticles in different medium, the density of functional modifications, the amount of ions released and so on. The variability of nanomaterial characterization in the relevant studies makes it difficult to compare and summarize. Faria et al. put forward a “minimum information reporting in bio-nano experimental literature” with regards to the details of the materials, cell or biological model, and experimental scheme in the study of nanomaterials and their biological interactions [160]. This standard is necessary to make the studies in this field more reliable and repeatable as well as to obtain a consensus on the characteristics of nanomaterials promoting angiogenesis.

### Differences in vitro cell models

Vascular endothelial cells are commonly used as cell models for angiogenesis in vitro. However, some endothelial cell lines, such as ECV304, do not fully possess the characteristics of endothelial cells and exhibit a poor capacity for tube formation in vitro. In addition, these cell lines are insensitive to some local microenvironment changes, which can lead to different conclusions.

The individual expression patterns of endothelial cells from different tissues are quite variable; for example, the Notch signalling of arterial endothelial cells is significantly higher than that of venous endothelial cells. Moreover, the expression of Notch ligand was higher in tumour vascular endothelial cells than in normal vasculature [161]. These findings provide not only clues for the differences in endothelial cell biological functions induced by nanomaterials in different vascular beds but also a research basis for the application of nanomaterials in normal and disease states.

MSCs can not only interact with endothelial cells but also promote vascular regeneration by differentiating into endothelial cells and vascular smooth muscle cells. There are few studies on whether nanomaterials are involved in induced directional differentiation. This may provide ideas for promoting early vascularization, but the combination of co-culture and complex microenvironments in vivo also makes the study more difficult.

According to the above mentioned minimum information reporting standards, we should also pay attention to more details of cell seeding in vitro, such as the cell source, cell seeding density or aging degree [160].

### Changes in the blood vessel permeability

Endothelial cells connect with each other by adherent junction and tight junction proteins, which perfectly balance the vascular barrier junction and selective permeation. It has been found that nanomaterials can disrupt vascular endothelial cadherin and open endothelial cell junctions, making their use a possible method for improving targeted delivery applications and bioavailability [162]. However, the destruction of the connection of endothelial cells by nanomaterials is also one of the effects that can affect vascular function and increase the toxic effects. Gold nanoparticles are not sensitive to HUVECs, although they can induce an endothelial cell gap [163]. Thus, it is worth exploring the mechanism of rearrangement of proteins comprising endothelial cell junctions. At present, the research on the barrier function of endothelial cells in the study of nanomaterials promoting angiogenesis, which is very important for the functional work of blood vessels, is less involved.

## Conclusion

The methods for improving scaffold materials include the discovery of new material components, the investigation of new manufacturing methods and the optimization of the biological properties of scaffold materials. Nanomaterials have unique structural properties, providing an innovative field for biomedical applications. Nanomaterials can not only directly affect the cytoskeleton and gene expression but also act as delivery vectors to enhance the sensitivity and targeting of angiogenic elements or growth factors. The application of nanomaterials has become an effective method to regulate the biological functions of cells. However, related research is still in the preliminary stages. Further systematic and standardized studies are needed to optimize nano-hierarchical structures for early angiogenesis, especially in complex microenvironments, which will provide reliable and effective evidences for clinical applications.

## Data Availability

Not applicable.

## Abbreviations

ECM: extracellular matrix
eNOS: endothelial nitric oxide synthase
ERK: extracellular regulated protein kinases
FAK: focal adhesion kinase
FGF: fibroblast growth factor
HIF: hypoxia inducible factor
HUVECs: human umbilical vein endothelial cells
hECFCs: human endothelial colony-forming cells
IL: interleukin
GO: graphene oxide
MSCs: mesenchymal stem cells
MAPK: mitogen-activated protein kinase
NO: nitric oxide
ROS: reactive oxygen species
TAZ: transcriptional coactivator with PDZ-binding motif
TFEB: transcription factor EB
VEGF: vascular endothelial growth factor
VEGFR: vascular endothelial growth factor receptor
YAP: yes-associated protein
